# Liver Proteome Profile of Growth Restricted and Appropriately Grown Newborn Wistar Rats Associated With Maternal Undernutrition

**DOI:** 10.3389/fendo.2021.684220

**Published:** 2021-05-28

**Authors:** Polyxeni-Maria Sarli, Antigoni Manousopoulou, Elias Efthymiou, Andreas Zouridis, Anastasios Potiris, Panagiota Pervanidou, Konstantinos Panoulis, Nikolaos Vlahos, Efthymios Deligeoroglou, Spiros D. Garbis, Makarios Eleftheriades

**Affiliations:** ^1^ Second Department of Obstetrics and Gynaecology, Medical School, National and Kapodistrian University of Athens, Athens, Greece; ^2^ Beckman Research Institute, City of Hope National Medical Center, Duarte, CA, United States; ^3^ First Department of Paediatrics, Medical School, National and Kapodistrian University of Athens, Athens, Greece; ^4^ Institute for Life Sciences, University of Southampton, Southampton, United Kingdom

**Keywords:** FGR, fetal programming, food restriction, metabolic disorders, liver proteomics

## Abstract

**Background:**

Fetal growth restriction (FGR) has been associated with adverse perinatal outcomes and epigenetic modifications that impact gene expression leading to permanent changes of fetal metabolic pathways and thereby influence development of disease in childhood and adult life. In this study, we investigated the result of maternal food restriction on liver protein expression in Wistar male newborn pups.

**Materials & Methods:**

Ten (n = 10) timed pregnant Wistar rats on their 14th day of gestation were randomly assigned to either control (n = 4) or food restricted group (n = 6). The control group had *ad libitum* access to food. In the food restricted group, maternal diet was limited in a moderate fashion (50%) from day 15 of pregnancy until delivery. All rats delivered spontaneously on day 21 and newborn pups were immediately weighed. Pups born to normally nourished mothers were considered as controls, while pups born to food restricted mothers were subdivided into two groups, based on their birth weight: growth restricted (FGR) and appropriately grown (non-FGR). Rats were euthanized immediately after birth and liver tissues of 11 randomly selected male offspring (FGR n = 4, non-FGR n = 4, control n = 3) were collected and analyzed using quantitative proteomics.

**Results:**

In total 6,665 proteins were profiled. Of these, 451 and 751 were differentially expressed in FGR and non-FGR vs. control, respectively, whereas 229 proteins were commonly expressed. Bioinformatics analysis of the differentially expressed proteins (DEPs) in FGR vs. control revealed induction of the super-pathway of cholesterol biosynthesis and inhibition of thyroid hormone metabolism, fatty acid beta oxidation and apelin liver signaling pathway. Analysis of DEPs in non-FGR vs. control groups showed inhibition of thyroid hormone metabolism, fatty acid beta oxidation, and apelin liver signaling pathway.

**Conclusion:**

This study demonstrates the impact of prenatal food restriction on the proteomic liver profile of FGR and non-FGR offspring underlying the importance of both prenatal adversities and birth weight on liver-dependent postnatal disease.

## Introduction

Fetal Growth Restriction (FGR) refers to a fetus that has failed to achieve its biological growth potential due to pathological conditions such as maternal/fetal disease and placental dysfunction. Fetal growth impairment is associated with perinatal morbidity and mortality, a 5- to 10-fold risk of *in utero* demise (1) and adverse neonatal outcomes (2). Furthermore, according to Barker’s hypothesis, an unfavorable intrauterine environment may have negative long-term effects in adult life (3). According to the thrifty phenotype hypothesis (4), FGR impairs the growth of organs such as the liver in order to maintain homeostasis of other crucial for survival organs and systems. These metabolic adaptations, enable fetuses to survive in a malnourished intrauterine environment. However, the cost of these adaptations is permanent physiological and epigenetic phenotypical alterations that are responsible for development of disease later in life such as obesity, diabetes, and cardiovascular disease (5).

Nutrition is one of the environmental variables with the widest range of effects on both physical growth and metabolism (6, 7). An expanding body of epidemiological evidence suggests that the nutritional environment experienced in fetal life increases the risk of chronic non-communicable diseases associated with human ageing. Maternal undernutrition constitutes a serious public health problem exhibiting large regional and within-country variations across the globe. Proper nutrition from preconception to delivery is critical for avoiding poor pregnancy and long-term outcomes for both the mother and child (8). A human model of fetal programming regarding the effects of maternal malnutrition on development of postnatal disease has been illustrated by the Dutch cohort from the Hunger Winter of 1944. It involved pregnant mothers exposed to famine and its long-term consequences for adult health. Poor maternal nutrition during gestation was associated with a higher prevalence of atherogenic lipid profile, obesity, insulin resistance, and cardiovascular disorders (9). To date, many experimental approaches have been designed to study the impact of FGR intervening either in maternal nutrition, placental blood flow or fetal wellness. Restricting maternal food intake is advantageous since it leads to an altered intrauterine nutritional milieu and growth impairment avoiding surgical intervention. Moreover, this type of animal model is closer to pregnancy malnutrition effects observed in humans (10, 11). Although a large number of animal models of FGR have investigated the impact of intrauterine environment on fetal epigenetic programming, there is little knowledge about the effects of maternal undernutrition on liver growth and physiology of appropriately grown (non-FGR) offspring of undernourished pregnancies.

Liver plays a major role in nutrients’ absorption and metabolism. During pregnancy, fetal growth restriction not only affects adversely liver’s growth but also its physiological function (12). Metabolic disorders namely, reduced oxidative phosphorylation, impaired mitochondrial function, antioxidant capacity, and altered nutrient metabolism are commonly found in FGR livers (13–15). It has been demonstrated that liver of FGR offspring seems to have an abnormally increased rate of gluconeogenesis contributing to insulin resistance and hyperglycemia (16, 17). Nevertheless, the exact mechanisms which are responsible for alterations in development, growth, and liver function leading to hepatic diseases are not adequately described. Previous animal studies have shown that maternal undernutrition and consequent FGR alters effectively the liver proteome through altered activities of many key enzymes (18). Proteomic studies in piglets revealed that many liver proteins involved in oxidative stress, intermediate metabolism, cell structure, and growth were differentially expressed in FGR offspring. Furthermore, nutritional models of fetal programming indicated that caloric restriction and low birth weight are strongly related with epigenomic changes in liver leading to insulin resistance and NAFLD (19). Our aim was to investigate the impact of maternal food deprivation on liver proteomic profile in three groups of newborn male Wistar rats: a) offspring of mothers that received standard laboratory diet (control group), b) offspring of food restricted mothers with low birth weight (FGR group), and c) appropriately grown offspring of food restricted mothers (non-FGR group).

Furthermore, the aim of this study was to examine whether prenatal food restriction during late gestation affects offspring liver proteome irrespective of birth weight and propose possible underlying pathophysiological mechanisms of liver fetal programming.

## Materials and Methods

### Animal Model

**Figure 1:**
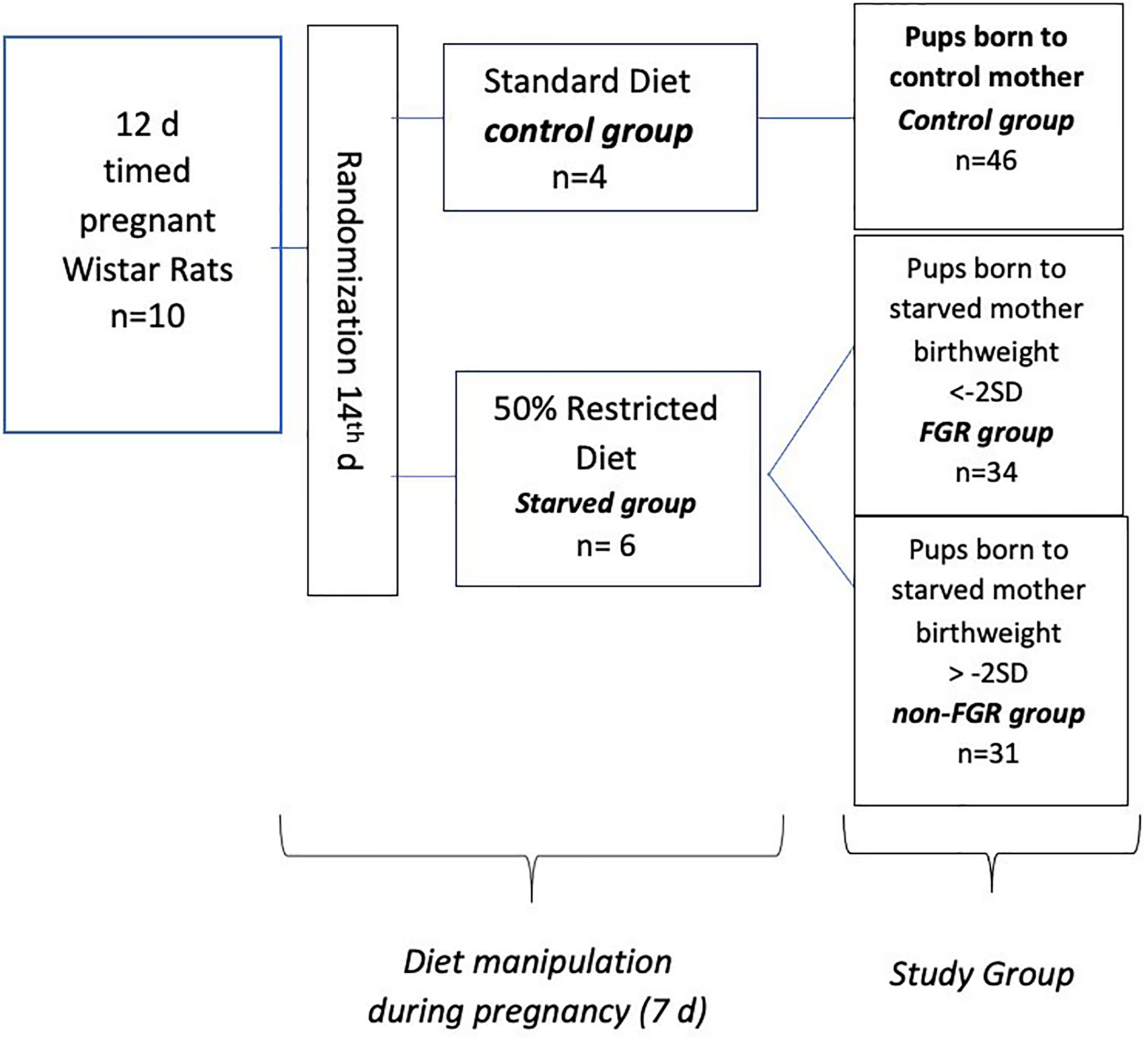
Experimental design of the study.

Ten (n = 10) timed pregnant Wistar rats, on their 12th day of gestation (Janvier Labs – Rodent research models & associated services, France), were hosted individually in 36 × 20 × 14 cm breeding boxes at the Laboratory of Experimental Surgery of the Second Department of General Surgery at Aretaieion Hospital, National and Kapodistrian University of Athens, Athens, Greece. Animals were housed under standard laboratory conditions (temperature between 22°C and 23°C, humidity 55% to 65% and 12-h light/dark cycles). All animals were fed with standard formula diet containing 18.5% protein (Mucedola S.r.l., Settimo Milanese, Italy) with ad libitum access to food and water as well, until day 14. Following randomization, pregnant dams were assigned to starved group (n = 6, diet restricted by 50%) and control group (n = 4 ad libitum access to food). Both groups had free access to fresh water. Control group’s food intake was measured on a daily basis. During the experimental period (from day 15th onward), rats of the starved group, were given half the amount of food that was on average consumed by the control group, based on measurements taken place the day before. Food restriction of the starved group lasted from 15th gestational day to delivery. All rats delivered spontaneously on the 21st gestational day and neonates were immediately weighted (**Figure 1**). Starved group’s offspring were categorized according to their birth weight as FGR (birth weight < mean birth weight of control group’s offspring − 2 × standard deviation) and non FGR (birth weight > mean birth weight of control group’s offspring − 2 × standard deviation) as previously described (20–24). Immediately after delivery, offspring were separated from their mothers and weighted. Neonates were anesthetized using inhaled sevoflurane, and euthanized. Liver tissues were rapidly removed. The time interval between rat’s sacrifice and specimens’ storage at −80°C did not exceed 15 min. All liver tissues were cleaned from blood with PBS (phosphate buffered saline). Specimens were stored at −80°C and sent packed in dry ice to the Centre for Proteomic Research, Institute for Life Sciences, University Southampton for proteomic analysis. Growth characteristics of mothers, gestation duration, litter size, birth weight of the pups, and organ weight were compared using the independent-samples t-test (IBM SPSS Statistics 22.0). Statistical significance was considered at p < 0.05. The animal model and study design have been previously presented and data on heart and brain proteomic analysis have been published (22, 24).

This study received ethics approval by the Ethics Committee of Aretaieion University Hospital, Medical School of the National and Kapodistrian University of Athens with registration number B 207/13-10-2016. Research license and approval for experimental animal (RjHan : WI – Wistar rats) utilization was granted by the Division of Agriculture and Veterinary Policy, District of Attica, Greece (Decision 5035/21-09-2017 and its modification 1211/19-03-2018). Animal handling was performed in accordance with the local applied laws (1197/1981 and 2015/1992) for the protection of animals and the Directive 2010/63/EU of the European Parliament and Council regarding the protection of animals used for research purposes. Based on the Directive 2010/63/EU of the European Parliament and Council, stating that animals should experience the minimum pain, suffering, and distress, we used inhalant sevoflurane in overdose as a euthanasia method.

### Quantitative Proteomics

Each liver tissue was dissolved in 200 μl of 0.5 M triethylammonium bicarbonate, 0.05% sodium dodecyl sulphate and homogenized using the FastPrep^®^-24 Instrument (MP Biomedicals, Santa Ana, CA, USA). Lysates were subjected to pulsed probe sonication (Misonix, Farmingdale, NY, USA) and centrifuged (16,000 g, 10 min, 4°C). The supernatant had been measured for its protein content using the Direct Detect^™^ system (Merck Millipore, Darmstadt, Germany). From each lysate, 100 μg of protein subjected to reduction, alkylation, trypsin proteolysis, and 11-plex TMT labelling according to manufacturer’s instructions. The resulting TMT peptides were initially fractionated with alkaline C_4_ reversed phase (RP) liquid chromatography. Each peptide fraction further separated with on-line nano-capillary C_18_ reverse phase liquid chromatography under acidic conditions, subjected to nanospary ionization, and measured with ultra-high resolution mass spectrometry using the hybrid ion-trap/FT-Orbitrap Elite platform. The unprocessed raw data files were submitted to Proteome Discoverer 1.4 for target decoy searching with SequestHT against the TREMBL Uniprot database for Rattus norvegicus (release date: January 2018). Reporter ion ratios derived from unique peptides only were used for the relative quantitation of each respective protein. Quantification ratios were median-normalized and log_2_ transformed. The threshold of percent co-isolation excluding peptides from quantification was set at 50. A one-sample T-Test was performed to identify proteins that were differentially expressed in the tissue from FGR and non-FGR compared to control rats. The two-stage step-up method of Benjamini, Krieger, and Yekutieli was used for multiple hypothesis correction. A *q-value* ≤ 0.05 was considered significant. Proteomics data have been deposited to the ProteomeXchange Consortium *via* the PRIDE partner repository.

## Results

### Experimental Model

Our experimental study consists of 111 newborn pups, which are divided into two sub-groups; the starved and the control group (food restricted group vs. control group; n = 65, 58.6% *vs.* n = 46, 41.4%), 57 offspring (51.4%) were male (22 in the control group and 35 in the food restricted group) and 54 (48.6%) were female (24 and 30), respectively. There was no statistical difference in post-delivery maternal bodyweight in both diet groups (control: 265 ± 25 gr, starved 270 ± 20 gr p = 0.769). Control group mothers (ad libitum food access) gave birth to control pups with mean body weight of 6.419 gr (SD: 0.436). The mean birthweight of the food restricted group was 5.423 gr, significantly different compared to controls (5.423 ± 0.610 gr *vs.* 6.419 ± 0.436 gr; p<0.001). Male neonates were heavier compared to females in the control group (6.659 g *vs.* 6.2 g, p < 0.001) but there was no statistically significant difference between them in the starved group (p = 0.666). Newborn pups delivered by starved mothers, were further divided to Fetal Growth Restricted (FGR) group when birthweight was < - 2SDs of the mean BW of the control offspring and non-FGR group when birthweight was > - 2SDs of the mean BW of the control. The cut-off between FGR and non-FGR neonates was set at 5.547 gr according to the aforementioned definition. Furthermore, there was statistically significant birthweight difference between FGR (4.796 gr ± 0.479 gr) and non-FGR (5.914 gr ± 0,479 gr) groups (p<0,001). Food restricted group reveals a remarkable sex differentiation impact on birth weight.

Even though male pups were heavier at birth compared to females in both the control group (control males vs. control females; 6.659 ± 0.324 g *vs.* 6.200 ± 0.413 g; p<0.001) and the non-FGR group (non-FGR males *vs.* non-FGR females; 5.930 ± 0.298 g. *vs.* 5.880 ± 0.131 g; p = 0.519), FGR male newborns weight 8.5% less than the female ones (FGR males *vs.* FGR females; 4.739 ± 0.629 g *vs.* 5.142 ± 0.240 g; p<0.05). Following this observation and in order to avoid bias due to sex differentiation we decided to include only male offspring for quantitative proteomic analysis.

Although liver weight of the non-FGR group was statistically significant higher compared to FGR pups (0.211 ± 0.047 *vs.* 0.280 ± 0.073, p < 0.0014), there was no difference in the liver weight to body weight ratio between groups (0.04274 ± 0.00743 *vs.* 0.04721 ± 0.01220, p = 0.10337) (**Tables 1**, **2**).

**Table 1:** Mean values for all experimental outcomes and comparisons between study groups.

	Group	Mean	p-value
***Length of gestation (days)***	***Starved***	21.22 ± 0.47	0.081
	***Control***	20.73 ± 0.06	
***Litter size (pups) (n = 111)***	***Starved***	10.83 ± 1.72	0.530
	***Control***	11.50 ± 1.29	
***Post-delivery maternal weight(g) (n = 10)***	***Starved***	268.83 ± 26.75	0.304
	***Control***	264.50 ± 10.47	
***Birth weight (g)***	***Starved***	5.423 ± 0.610	<0.001
	***Control***	6.419 ± 0.436	
***Liver weight (g)***	***Starved***	0.245 ± 0.070	0.117
	***Control***	0.266 ± 0.057	
	***Fgr***	0.211 ± 0.047	<0.001
	***non-Fgr***	0.280 ± 0.073	
***Liver to body weight (g)***	***Starved***	0.04498 ± 0.01026	0.112
	***Control***	0.41753 ± 0.00946	
	***FGR***	0.04274 ± 0.00743	0.103
	***non-FGR***	0.04721 ± 0.00743	
***Brain weight (g)***	***Starved***	0.150 ± 0.045	<0.001
	***Control***	0.180 ± 0.044	
	***FGR***	0.151 ± 0.058	0.783
	***non-FGR***	0.148 ± 0.043	
***Brain to body weight (g)***	***Starved***	0.02826 ± 0.00968	0.905
	***Control***	0.02806 ± 0.00598	
	***FGR***	0.03157 ± 0.01093	0.009
	***non-FGR***	0.02496 ± 0.00698	

**Table 2:** Birth and liver tissue mean weights of the newborn pups in control, food restricted group and both subcategories of starved group.

	Control Group	Starved Group	FGR	Non-FGR
***Birth weight***				
Male (n = 57)	6.659 ± 0.324	5.454 ± 0.744*	4.739 ± 0.629*	5.930 ± 0.298*
Female (n = 54)	6.200 ± 0.413	5.388 ± 0.410*	5.388 ± 0.410*	5.880 ± 0.131**
***Liver weight***				
Male	0.296 ± 0.028	0.240 ± 0.077*	0.173 ± 0.038	0.291 ± 0.056
Female	0.271 ± 0.059	0.250 ± 0.061*	0.222 ± 0.030	0.3 ± 0.069
***Liver to body weight***				
Male	0.05911 ± 0.08464	0.04438 ± 0.00453*	0.04311 ± 0.00765	0.04929 ± 0.00905
Female	0.04407 ± 0.01095	0.04594 ± 0.00941**	0.04303 ± 0.00519	0.051 ± 0.01212

*p-value < 0.001, **p-value < 0,01 (compared to control group).

### Proteomic Analysis

**Figure 2:**
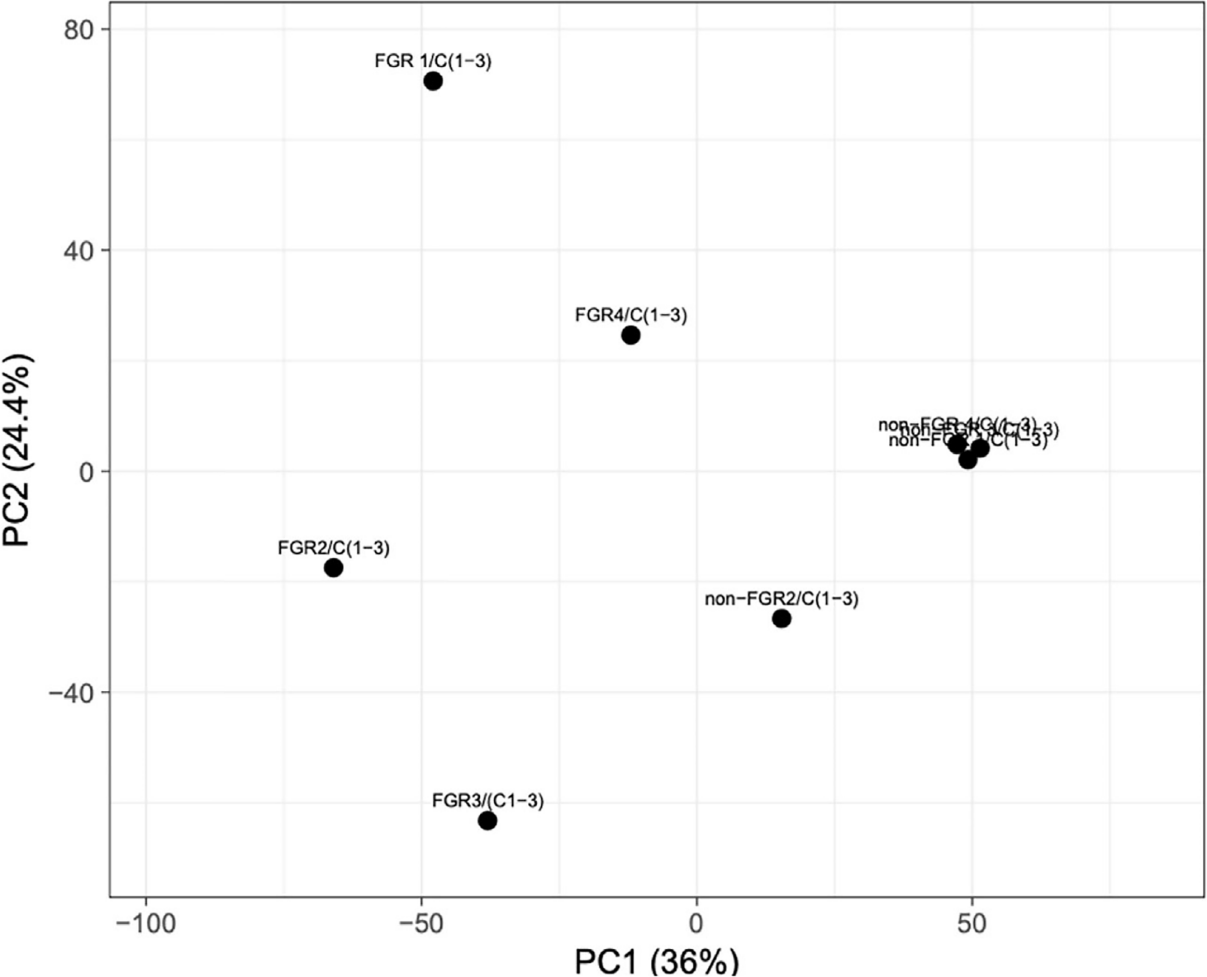
Principal component analysis (PCA) of all quantified proteins revealed that liver of fetal growth restricted pups had a heterogeneous proteomic profile compared to non-FGR ones.

**Figure 3:**
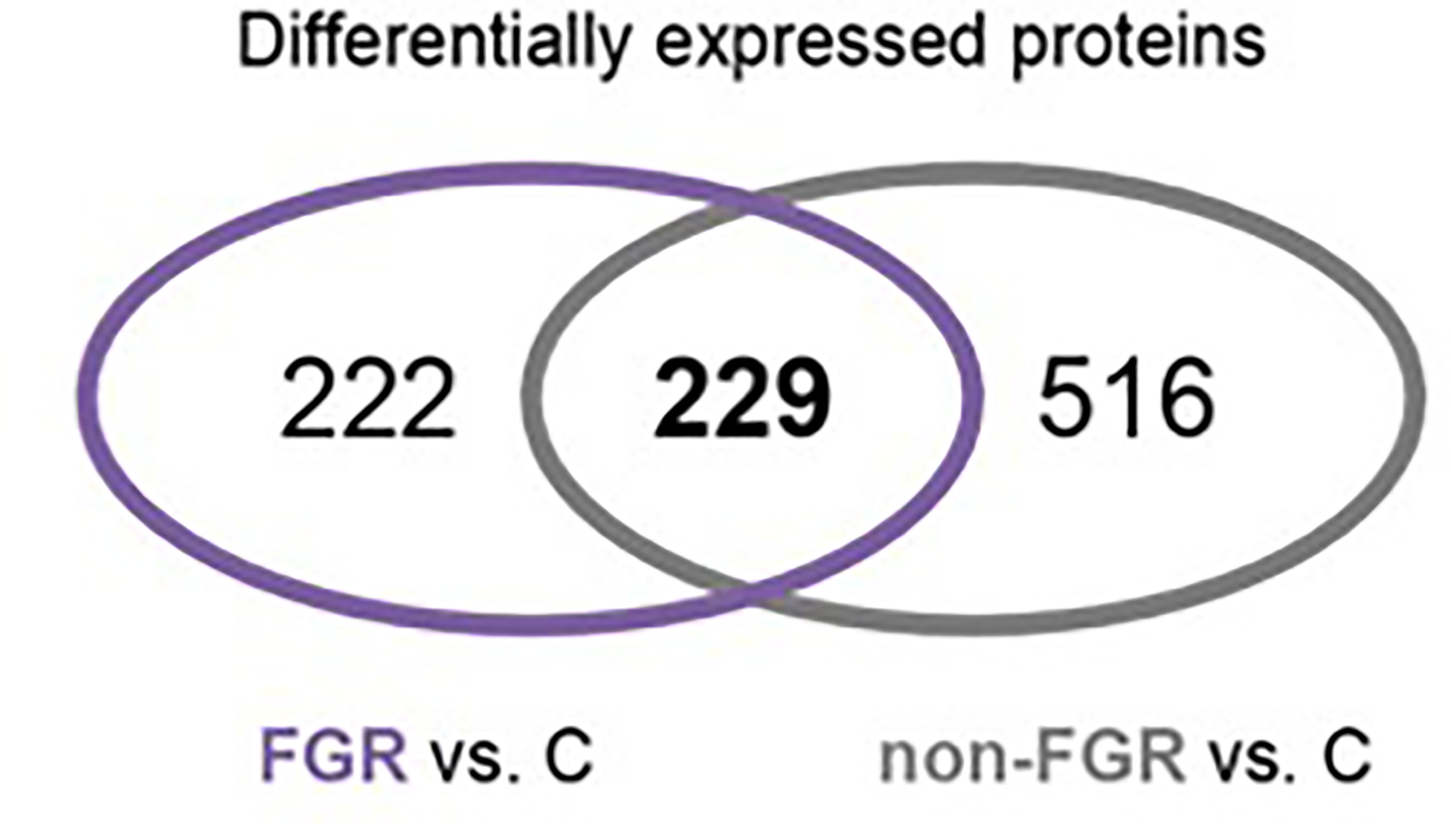
Venn diagram of common Differentially expressed proteins in FGR *vs.* Control and non-FGR *vs.* Control group.

**Figure 4:**
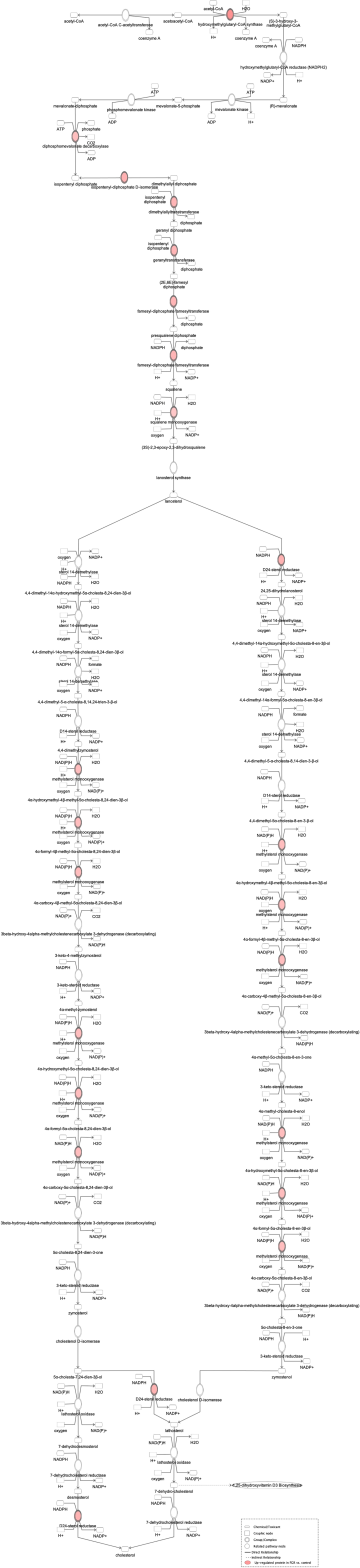
Ingenuity Pathway Analysis of DEPs between FGR *vs.* Control group. *Induction of the super pathway of cholesterol biosynthesis*. **(z = 2.2, p = 1.5e-4)**.

**Figure 5:**
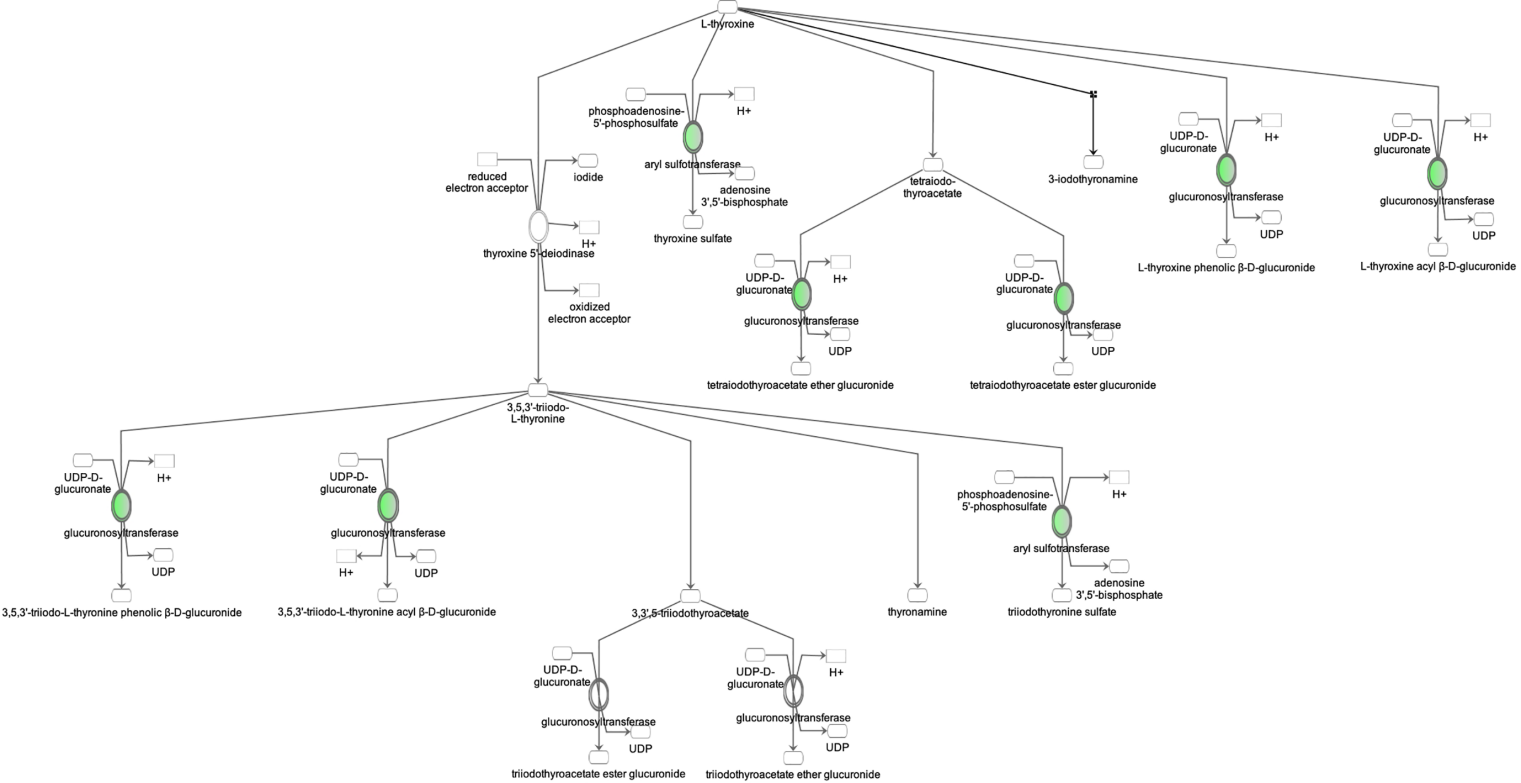
Ingenuity Pathway Analysis of DEPs between FGR vs. Control group. *Inhibition of thyroid hormone metabolism.* **(z = -2.0, p = 4.6e-3)**.

**Figure 6:**
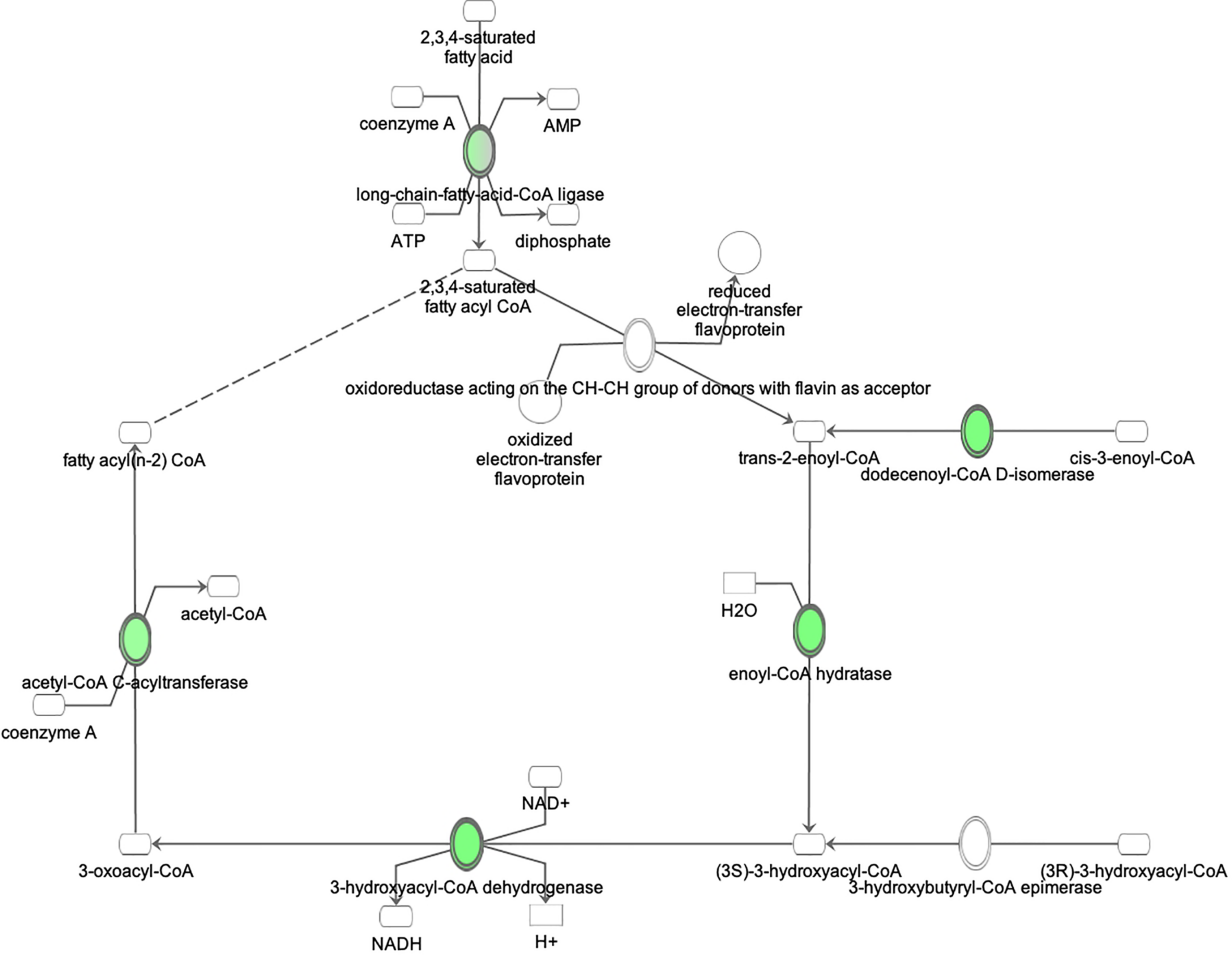
Ingenuity Pathway Analysis of DEPs between FGR *vs.* Control group. *Inhibition of fatty acid beta oxidation*. **(z =** −**2.0, p = 2.7e-3)**.

**Figure 7:**
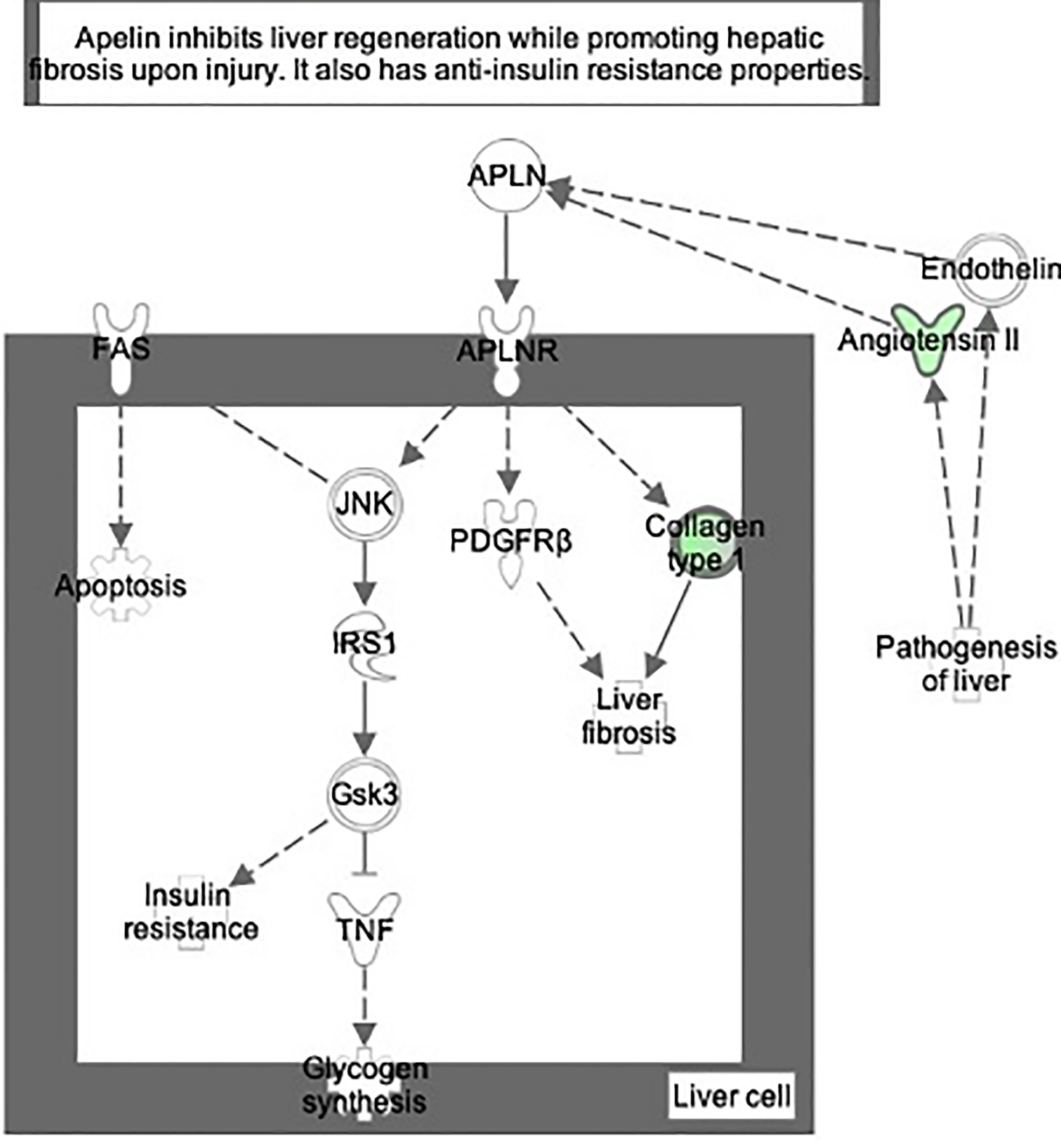
Ingenuity Pathway Analysis of DEPs between FGR vs. Control group. *Inhibition of apelin liver signaling pathway.* **(z = −2.2, p = 8.5e-5)**.

**Figure 8:**
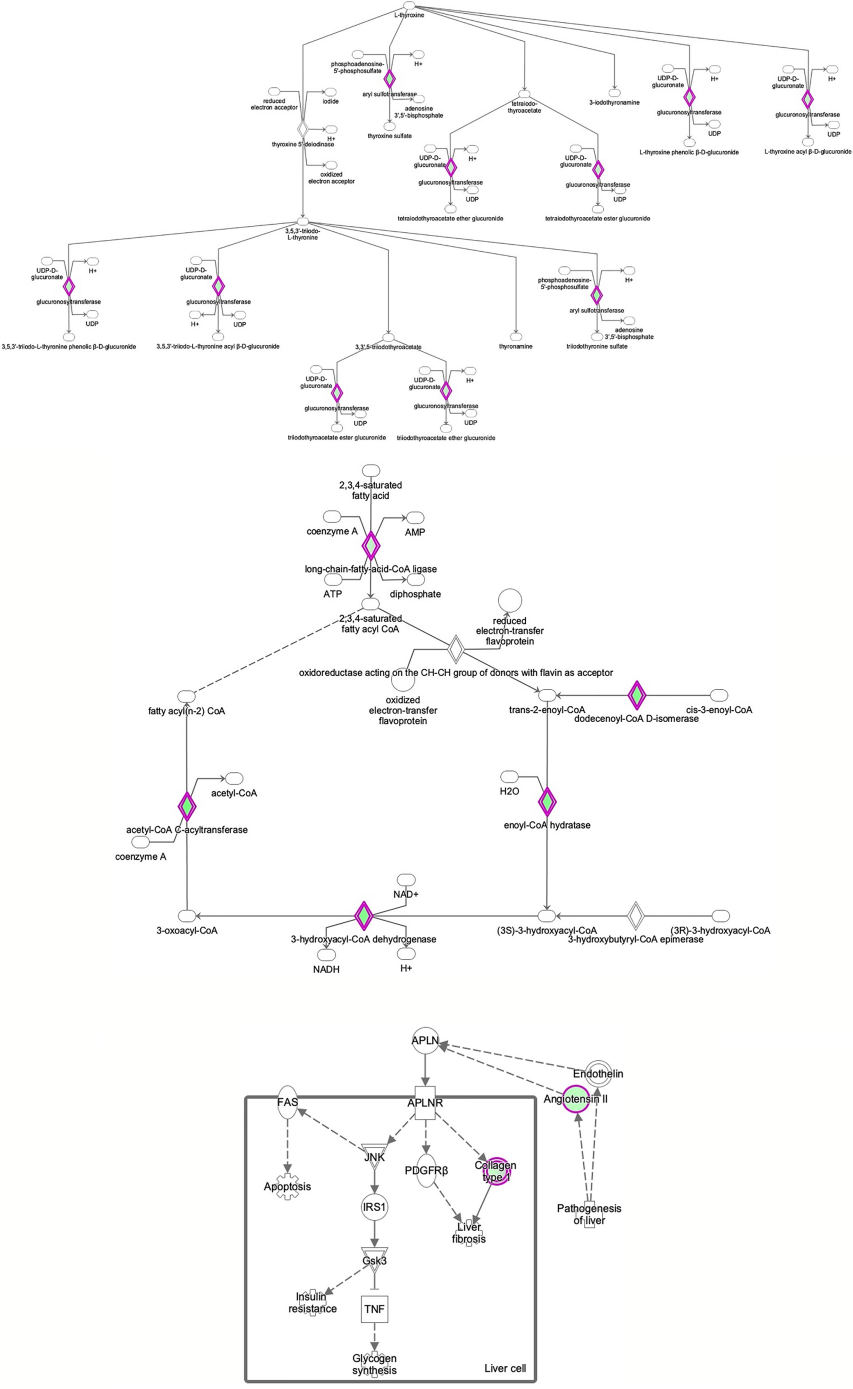
Ingenuity Pathway Analysis of differentially expressed proteins in non-FGR compared to control group showed *inhibition of thyroid hormone metabolism* **(z=** −**2.0, p = 2.5e-2)**, *fatty acid beta oxidation* **(z =** −**2.0, p = 1.6e-2)** *and apelin liver signaling pathway* **(z = −2.0, p = 6.7e-3)**. Apelin inhibits liver regeneration while promoting hepatic fibrosis upon injury. It also has an anti-insulin resistance properties.

Proteomic analysis of male offspring livers ended up in the profiling of 6,665 proteins (peptide level q<0.05) (**Supplementary Table 1**). Among the quantified proteins, 451 proteins were differentially expressed in FGR *vs.* control (**Supplementary Table 2**) and 782 in non-FGR *vs.* control group (**Supplementary Table 3**). Of these, 76 were commonly up-regulated and 153 commonly down-regulated in both FGR and non-FGR compared to control (**Supplementary Table 4**) (**Figure 8**). Principal component analysis (PCA) of all quantified proteins showed a distinct proteomic liver profile of FGR compared to non-FGR rats (**Figures 2**, **3**). Bioinformatics analysis of differentially expressed proteins (DEPs) in FGR compared to control groups using Ingenuity Pathway Analysis (IPA) showed: a. induction of the super pathway of cholesterol biosynthesis (z = 2.2; p = 1.5e-4) (**Figure 4**), and b. inhibition of thyroid hormone metabolism (**Figure 5**) (z = −2.0; p = 4.6e-3), fatty acid beta oxidation (z = −2.0; p = 2.7e-3) (**Figure 6**), and apelin liver signaling pathway (**Figure 7**) (z = −2.2; p = 8.5e-5). Enrichment analysis of the DEPs in non-FGR vs. control groups using IPA showed: a. induction of immune cell adhesion (z = 2.9; p = 1.1e-7) and b. inhibition of thyroid hormone metabolism (z = −2.0; p = 2.5e-2), fatty acid beta oxidation (z= -2.0; p = 1.6e-2) and apelin liver signaling pathway (z = −2.0; p = 6.7e-3) (**Figure 8**).

## Discussion

Numerous studies have shown the impact of adverse early-life environment on disease during infancy, childhood, and adult life (25, 26). Fetal growth restriction is associated with significant perinatal and subsequent long-term morbidity and mortality (27). FGR neonates and infants demonstrate a variety of complications involving multiple organs and systems such as pulmonary, gastrointestinal, immune, and central nervous system. Regarding the endocrine system, FGR is associated with altered glucose metabolism, transiently low thyroxin levels and cortisol deficiency. Furthermore, FGR programs both childhood and adult disease, associated with increased risk of obesity, insulin resistance, non-alcoholic fatty liver disease (NAFLD) and cardiovascular disease (13, 28, 29).

Using a well-defined FGR rat model, this study shows that maternal food restriction plays a crucial role, impairing liver intrauterine growth and altering its proteomic expression. In our study liver weight was reduced in proportion to body weight in FGR compared to non-FGR pups. On the contrary, brain weight did not differ significantly between the abovementioned two groups (**Table 1**) indicating a late-onset FGR model resembling to the commonest FGR phenotype in human population (30). This study aimed to a better understanding of the proteomic mechanisms of liver developmental dysfunction induced by prenatal food restriction investigating possible differentiations in liver proteomic expression in both growth restricted (FGR) and appropriately grown (non-FGR) offspring born to starved mothers. To our knowledge, this study is the first one to report the proteomic profiling of liver in both FGR and non-FGR Wistar rat offspring exposed to prenatal food restriction. Our study demonstrated that maternal undernutrition produced a distinct proteomic profile in FGR and non-FGR pups. These changes are indicative of an induction of cholesterol biosynthesis and inhibition of thyroid hormone metabolism, fatty acid beta oxidation, and apelin liver signaling

Bioinformatic analysis of DEPs in the FGR group vs. control showed induction of cholesterol biosynthesis. Regarding cholesterol biosynthesis, metabolomic studies have shown that FGR fetuses have higher concentrations of cholesterol such as VLDL and LDL, lipoproteins, and triglycerides (31). Lipids are vital molecules for life, providing energy for metabolic processes. Furthermore, cholesterol is a key element for brain neurodevelopment and a precursor of many hormones like sex steroids (32, 33). Fetal liver is the main source of circulating lipoproteins, as in adults. Alterations of VLDL concentrations, which are mostly synthesized in fetal liver, imply an altered hepatic synthesis of lipoproteins caused by FGR. Remarkably, the lipid profile of FGR fetuses resembles to adults presenting with atherosclerosis and dyslipidemia (34, 35).

The apelin signaling pathway, thyroid metabolism, and fatty acid beta oxidation were inhibited in both FGR and non-FGR neonate rats, indicating these might be a result of maternal undernutrition regardless the fetus’ growth. Apelin is a regulatory peptide and in conjunction with its receptor, are both expressed in a wide range of tissues such as central nervous system, heart, and liver. Apelin is also produced by adipocytes and latest studies proposed its crucial role in energy metabolism and enhancement of insulin sensitivity (36). Our study in accordance with previous ones, have showed inhibition of apelin signaling and reduced plasma concentrations as a potential response to undernutrition (37). Recent studies have highlighted the paramount importance of apelin and its receptor, since they have been proposed as a valuable new treatment target in type 2 diabetes (38, 39).

Our study showed that in both FGR and non-FGR offspring of calorie restricted mothers, liver thyroid hormones’ metabolism is inhibited. Thyroid hormones play a key role to thermoregulation, specifically in norepinephrine (NE) controlled thermogenesis (40). Brown adipose tissue thermogenic activity which is triggered by NE is under triiodothyronine (T_3_) control (41). Low T3 plasma levels are associated with impaired thermogenesis and predisposition to diet-induced obesity in neonatal and adult life despite later normalization of T_3_ plasma concentrations (42, 43). Hypothermia and transiently low thyroxine levels are common neonatal complications of FGR however no information is available in appropriately grown neonates born to undernourished mothers (44).

Our model suggests inhibition of fatty acid metabolism not only in FGR liver but in non-FGR liver as well. Liver is the central organ of fatty acid metabolism. Both obesity and insulin resistance are closely related with disrupted fatty acid metabolism (26). Inhibition of this metabolic process leads to non-alcoholic fatty liver, liver steatosis, and subsequent insulin resistance deterioration. In a previous study of our team where NEFA (Non-Esterified Fatty Acids) concentrations were compared between FGR and non-FGR rats at one year of age there was no statistical difference between groups. It seems that food restriction produces the same adipose tissue response in both the FGR and non-FGR groups, suggesting that it is the adverse prenatal event that determines certain metabolic profiles rather than birthweight (45).

During the last few decades, a remarkable increase in the prevalence of non-alcoholic fatty liver (NAFLD) in modern western world has been observed (8). Various risk factors namely obesity, insulin resistance or overt diabetes, dyslipidemia, and metabolic syndrome are potential precursors of NAFLD. As non-alcoholic fatty liver seems to be the major cause of chronic liver disease, it is important to recognize individuals with increased risk for this condition, such as FGR offspring, in order to intervene early and prevent its pathogenesis. Recent studies have revealed that NAFLD affects low birth weight offspring even during childhood (46, 47). Although increased hepatic lipids promote insulin resistance, the exact mechanism through which early insulin resistance accelerates the development of non-alcoholic fatty liver in FGR individuals remains unclear (28, 48, 49).

To date, few studies have investigated the proteomic profile of the placenta of FGR human offspring. Bioinformatic analysis of differentially expressed proteins of FGR placentas revealed a distinct proteomic network associated with growth restriction. Most of these studies showed upregulation of proteins related to oxidative stress, cellular apoptosis, inflammation, and intracellular lipid metabolism (50). Moreover, Chassen et al. demonstrated increased expression of two fatty acid transport proteins and seven long chain fatty acids in the cellular triglyceride fraction in placentas of FGR fetuses compared to appropriately grown. These results establish some additional adaptive mechanisms of growth restricted fetuses in order to survive in this adverse intrauterine environment (51).

## Conclusion

According to fetal programming theory, fetal malnutrition induces adaptive processes that permanently change growth, physiology, and metabolism of the offspring. Maternal undernutrition alters the proteomic profile of the neonatal liver which is a key organ of many metabolic processes supporting homeostasis. In our study, FGR (representing a model of human neonates with growth restriction) and non-FGR pups (representing a model of human infants having experienced adverse intrauterine conditions but born with normal body weight) have developed both common and different metabolic phenotypes. Thus, suggesting that both intrauterine adversities and birthweight determine the metabolic profile of the offspring. This study contributes to a better understanding of the proteomic mechanisms of liver developmental dysfunction induced by prenatal food restriction and helps to explain the intrauterine origin of adult metabolic disease. Research in both animal and humans should focus on early detection of possible pregnancy complications and adequate prevention and intervention strategies as well, in order to promote postnatal health and ameliorate diseases with developmental origins, such as non-communicable diseases.

## Study Limitation

The extrapolation of our results to human population should be made with caution as in all experimental studies. The use of specific animal model of prenatal malnutrition, the number of animals in each experimental group that should be kept to the minimum and differences between human physiology and disease that are not adequately captured by animal models may limit the strength of our findings.

## Data Availability Statement

The datasets presented in this study can be found in online repositories. All proteomic data are uploaded at the ProteomeXchange Consortium via the PRIDE partner repository (dataset identifier PXD011406) http://proteomecentral.proteomexchange.org/cgi/GetDataset?ID=PXD011406.

## Ethics Statement

This animal study was approved by the Ethics Committee of Aretaieion University Hospital, Medical School of the National and Kapodistrian University of Athens with registration number: B 207/13-10-2016 and the Directorate of Veterinary Services (protocol number: 1211/19-03-2018).

## Author Contributions

Conceptualization, ME; formal analysis, P-MS, AM, AP, AZ, and EE; funding acquisition, P-MS and ME; investigation, P-MS, AM, AP, AZ, and ME; methodology, P-MS, AM, SDG, and ME; project administration, ME; resources, ME and SDG; supervision, KP, PP, NV, SDG, and ME; visualization, P-MS and AM; writing—originaldraft, P-MS and AM; writing— review and editing, AM, KP, ED, NV, PP, SDG, and ME. All authors contributed to the article and approved the submitted version.

## Funding

This research project was supported in part by Procter & Gamble Hellas “George Papanicolaou 2018–2020” research grant. The funder was not involved in the study design, collection, analysis, interpretation of data, the writing of this article, or the decision to submit it for publication.

## Conflict of Interest

SG is Founder, President, and CEO of Proteas Bioanalytics Inc., BioLabs at the Lundquist Institute, 1124 West Carson Street, MRL Building, 3rd Floor, Torrance, CA 90502.

The remaining authors declare that the research was conducted in the absence of any commercial or financial relationships that could be construed as a potential conflict of interest.

## Supplementary Material

Supplementary Table 1 All identified proteins (peptide FDR p<0.05) by the proteomic analysis.

Supplementary Table 2 Differentially expressed proteins (DEPs) in FGR vs. control.

Supplementary Table 3 Differentially expressed proteins (DEPs) in non-FGR vs. control.

Supplementary Table 4 Commonly up- or down-regulated proteins in both FGR and non-FGR vs. controls.
